# A One Health Evaluation of the Surveillance Systems on Tick-Borne Diseases in the Netherlands, Spain and Italy

**DOI:** 10.3390/vetsci9090504

**Published:** 2022-09-14

**Authors:** Aitor Garcia-Vozmediano, Daniele De Meneghi, Hein Sprong, Aránzazu Portillo, José A. Oteo, Laura Tomassone

**Affiliations:** 1 Department of Veterinary Sciences, University of Turin, L. go Braccini, 2, 10095 Grugliasco, TO, Italy; 2 Network for EcoHealth and One Health (NEOH), European Chapter of Ecohealth International, Kreuzstrasse 2, P.O. Box, 4123 Allschwil, Switzerland; 3 Centre for Infectious Disease Control, National Institute for Public Health and the Environment (RIVM), Antonie van Leeuwenhoeklaan 9, 3720 MA Bilthoven, The Netherlands; 4 Center of Rickettsiosis and Arthropod-Borne Diseases (CRETAV), Department of Infectious Diseases, San Pedro University Hospital-Center for Biomedical Research of La Rioja (CIBIR), Calle Piqueras 98, 26006 Logroño, La Rioja, Spain

**Keywords:** One Health Evaluation, surveillance, tick-borne diseases, transdisciplinary collaboration, NEOH framework

## Abstract

**Simple Summary:**

Ixodid ticks and tick-borne diseases are expanding their geographical range, but surveillance activities vary among countries. We analysed the surveillance systems in place in the Netherlands, Spain and Italy, to identify ideal elements to monitor tick-borne diseases, by using a One Health evaluation protocol. We identified differences among the three surveillance systems, with the Dutch initiative showing a high level of transdisciplinary collaboration, good identification of the actors and engagement of the public in research and education. Measurable outcomes have been generated, such as the reduction in tick bites and the discovery of new pathogens and tick species. In Italy and Spain, surveillance systems are based on compulsory notification to health authorities; legislation seems relevant but law enforcement alongside the availability of economic resources is rather fragmented and limited to the most severe diseases. The non-scientific community is marginally considered and collaborations are limited to local initiatives. Research activities in both countries have mostly contributed to gaining knowledge on the distribution of tick species and the discovery of new pathogens. Although all TBD surveillance plans comply with the EU regulations, the initiatives characterised by trans-disciplinary collaboration may be more effective for the surveillance and prevention of tick-transmitted diseases.

**Abstract:**

To identify ideal elements for the monitoring and prevention of tick-borne diseases (TBD), we analysed the surveillance systems in place in the Netherlands, Spain and Italy. We applied a semi-quantitative evaluation to identify outcomes and assess the degree of One Health implementation. Differences emerged in the surveillance initiatives, as well as the One Health scores. The Dutch surveillance is dominated by a high level of transdisciplinary and trans-sectoral collaboration, enabling communication and data sharing among actors. Different project-based monitoring, research and educational activities are centrally coordinated and the non-scientific community is actively involved. All this yielded measurable health outcomes. In Italy and Spain, TBD surveillance and reporting systems are based on compulsory notification. Law enforcement, alongside dedicated time and availability of economic resources, is fragmented and limited to the most severe health issues. Veterinary and human medicine are the most involved disciplines, with the first prevailing in some contexts. Stakeholders are marginally considered and collaborations limited to local initiatives. Research activities have mostly contributed to gaining knowledge on the distribution of tick vectors and discovery of new pathogens. Although all TBD surveillance plans comply with EU regulations, initiatives characterised by transdisciplinary collaboration may be more effective for the surveillance and prevention of TBD.

## 1. Introduction

The risk of introduction and spread of emerging pathogens of medical and veterinary concern has been increasing in Europe. Tick-borne diseases (TBD), in particular, are highly sensitive to changes in weather and climate, which have consequently led to a substantial rise in their incidence linked to the expanding distribution of their tick vectors [1]. Hence, tick-borne zoonoses such as Lyme borreliosis (LB), tick-borne encephalitis (TBE) and Crimean-Congo haemorrhagic fever (CCHF) are, among others, emerging health problems that increasingly threaten to become endemic in new areas.

According to European Union (EU) legislation, EU-member states have the obligation to notify some infectious diseases to the national authorities and/or the European Commission (Decision No. 2000/96/EC). EU policies about communicable diseases aim at disease monitoring, early detection and rapid response. In 1999, a network for epidemiological surveillance and disease control was established to promote the cooperation and coordination among member states (Decision No. 2119/98/EC). Within this framework, surveillance activities are carried out in the fields of human and animal health to collect epidemiological data at country level, while promoting the implementation of specific surveillance plans for disease prevention, control and early detection. At present, the European Centre for Disease Prevention and Control (ECDC) and the European Food Safety Authority (EFSA) jointly coordinate a specific programme on vector-borne diseases and provide scientific advice to reduce the risk to animals and humans [2]. In the past years, EU-legislation on communicable diseases was amended to include the surveillance of some TBD. The Commission Decision No. 2000/96/EC embraced the viral haemorrhagic fevers, including CCHF, under the category of ‘serious imported diseases’. Later, in 2012, the Commission Decision No. 2012/492/UE specifically incorporated the section of ‘Tick-borne diseases’, with reference to TBE. More recently, this list has been updated with the definition of disease cases for some emerging and re-emerging vector-borne diseases, including Lyme neuroborreliosis (Commission Implementing Decision 2018/945). 

Tick-borne diseases represent a special challenge for European surveillance systems and national health authorities due to the complex epidemiology, difficulties in their diagnosis (sometimes needing the awareness of open-minded physicians) and because TBD prevention mainly relies on public health education. The surveillance of TBD can benefit from cooperation among several disciplines, including entomology, ecology, veterinary and human medicine, among others, to monitor tick distribution, animal reservoirs and disease cases in humans and also in domesticated animals [3,4] within an integrated, One Health (OH) approach. 

Within the framework of existing research collaborations on tick-borne diseases among the authors, we evaluated the surveillance systems on TBD in place in the Netherlands, Italy and Spain, with a focus on the degree of OH implementation in the initiatives. All three countries are experiencing changes in ticks/TBD epidemiology (e.g., emergence of CCHF in Spain, TBE in the Netherlands, and the increase in tick distribution range in Italy). For this purpose, we applied the semi-quantitative evaluation protocol developed by the EU COST Action TD1404 “Network of Evaluation of One Health” (NEOH)) [5], which enables us to develop a profound understanding of the strengths, weaknesses and gaps of established surveillance programmes [6]. 

## 2. Materials and Methods

### 2.1. Data Collection

To characterise the surveillance systems under review, we gathered data from different information sources, including legislation, surveillance reports at international, national and/or regional scales and the available scientific literature. These data were thoroughly analysed and discussed among the manuscript authors per each country, in order to describe the context, the health initiative and the theory of change. 

### 2.2. NEOH Evaluation Tool 

The evaluation method follows guidelines outlined in Rüegg et al. [7] and consists of a mixed methods approach, including a descriptive and qualitative assessment combined with a semi-quantitative scoring that measures the degree of OH that characterises the health initiative. It is based on a questionnaire that explores different operational (thinking, planning, working) and infrastructural (learning, sharing, systemic organisation) aspects within the initiative. OH thinking refers to the specific dimensions and scales involved and/or affected by the health issue, and how they match within the implemented initiative. OH planning assesses the resource allocation, suitability and adaptability of health programs to address common objectives, considering the integrated nature of the initiative. OH working focuses on the transdisciplinary approach and participatory engagement which relies on appropriate leadership and management. OH sharing evaluates the extent and methods of information and data sharing infrastructures within the initiative. OH learning examines knowledge exchange infrastructure and how this supports learning within the system and in the broader environment. Finally, the systemic organisation looks at the implementation of shared leadership and governance to promote engagement of all disciplines for effective teamwork. Questions related to all these OH aspects were scored with values between zero and one, where zero indicates the absence of the component within the initiative, and a value of one reflects its full implementation. Scores were inserted in a Microsoft Excel workbook, modelled on a template provided by Rüegg et al. [6]. Due to the complexity and fragmentation of the systems implemented in Spain and Italy, for these countries OH sharing was only quantitatively assessed and systemic organisation could not be evaluated. 

## 3. Results

### 3.1. Context and Description of the Systems and Health Initiatives

#### 3.1.1. The Netherlands

In the Netherlands, LB has been a growing health issue since the 1990s, with considerable societal cost. Indeed, it is estimated that LB affects around 27,000 people annually, with a cost of EUR 19.3 million for the Dutch healthcare [8,9]. Human cases have been increasing over the years, reaching a maximum incidence rate of 149 diagnosed *erythema migrans* per 100,000 inhabitants in 2017 [10]. Accordingly, the country has been experiencing a sharp increase in the number of medical consultations regarding tick bites [10,11,12]. 

Other emerging TBD have been receiving attention from Dutch health authorities in recent years. Tick-borne encephalitis was first discovered in 2016, circulating in both ticks and wild animals [13]; afterwards, the first autochthonous human cases were identified [14,15]. This viral disease might also impact the food chain, causing economic losses to Dutch animal producers and food industry, since the virus can be transmitted through the consumption of raw dairy milk and products. Sporadic reports of exotic tick vectors, such as *Hyalomma marginatum*, have additionally alerted the health authorities to the potential introduction of emerging pathogens, including CCHF virus and *Rickettsia aeschlimannii* [16,17]. 

According to the Dutch Public Health Act n. 461/2008, the control of infectious diseases corresponds to the municipalities, which are endorsed by 25 municipal health directorates. The National Institute for the Public Health and the Environment (RIVM) is a multidisciplinary health centre that serves as coordinating structure for the national health surveillance [18]. This institution holds the Centre of Infectious Disease Control (RIVM-CIb), which aims at the monitoring, prevention, control and management of infectious diseases. Moreover, RIVM-CIb supports and coordinates different control activities at the national and international level. At the national level, it provides advice and support to local and regional health services to promptly identify outbreaks of endemic and emerging infections. 

**Figure 1:**
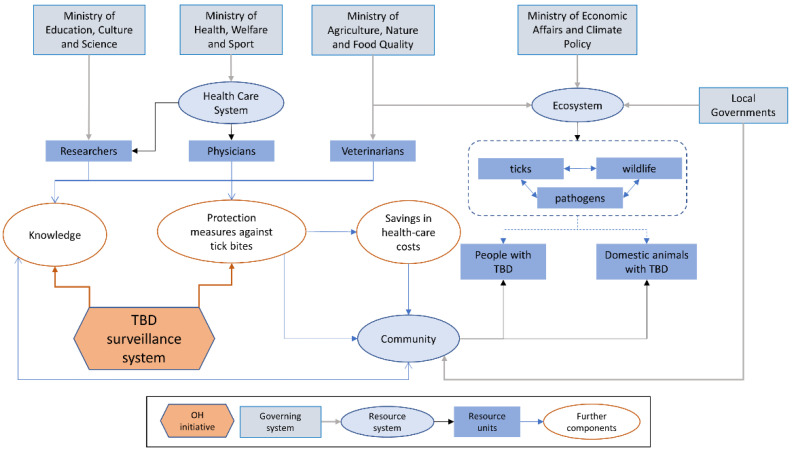
Visual representation of the TBD surveillance initiative in the Netherlands within its system. Relationships (arrows) are classified as governance (grey), membership (black), and causal interactions (blue). The red hexagon represents the initiative with arrows where it impacts the system. Note: TBD in pets and livestock are only partially considered by the initiative (modified from Rüegg et al., 2018).

Even though TBD are not notifiable diseases in the Netherlands, a surveillance system based on multiple projects has been implemented (Figure 1). RIVM, together with different health entities and universities, carries out several monitoring, research, education and control activities. A national awareness week is held annually during springtime to mark the onset of the tick season; during this week, information and advice about ticks and TBD are provided to the general public [19]. These information campaigns also include the development of online games, targeting school-aged children, videos [20] and a mobile application named ‘Tick radar’ [21]. This latter was launched in 2012 from the collaboration between RIVM and Wageningen University and provides information and advice to users, shows real-time maps illustrating the distribution of tick bites and LB cases based on users’ reports.

#### 3.1.2. Italy

**Figure 2:**
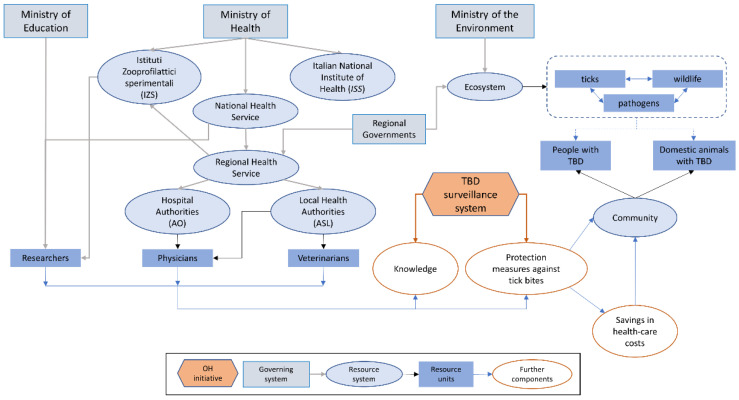
Visual representation of the TBD surveillance initiative in Italy within its system. Relationships (arrows) are classified as governance (grey), membership (black), and causal interactions (blue). The red hexagon represents the initiative with arrows where it impacts the system (modified from Rüegg et al., 2018).

In Italy, the National Health Service is coordinated by the Ministry of Health. However, the health system is highly decentralised, with most administrative and organisational powers governed by the 21 Regions (Figure 2). Regional Health Services comprise Local Health Authorities (ASL) and Hospital Authorities (AO). Regions may implement surveillance and have the obligation to report cases of notifiable diseases to the Ministry of Health. The Italian National Institute of Health (*Istituto Superiore di Sanità*, *ISS*) is the main Italian research institute in the biomedical and public health field and is the technical and scientific body of the National Health Service. Finally, ten Experimental Zooprophylactic Institutes (*Istituti zooprofilattici sperimentali*, *IZS*), distributed on the national territory, operate in concert with the Ministry of Health, regional veterinary services and the local health services to assure diagnostic activities, epidemiological surveillance, research and training.

National legislation requires the mandatory surveillance of some TBD. The still ongoing Ministerial Decree 15/12/1990 includes rickettsioses, Q fever and tularemia within ‘class 2’ diseases, for which the local health agencies and the regions are required to notify the Ministry of Health about the cases within 48 h. Lyme borreliosis is included within the ‘class 5’, for which the reporting of cases to the Ministry of Health is performed yearly. The Directive 2003/99/EC on the monitoring of zoonoses and zoonotic agents was implemented in the Italian context with the Legislative Decree 191/2006, which enables the regions to carry out specific LB monitoring according to the epidemiological situation. 

The Ministry of Health Circular No. 17500, 2018 establishes the national surveillance and response plan for TBE, specifying the mandatory reporting of disease cases within 24 h to the local health services; in case of confirmation, these must be communicated to the Ministry of Health within 12 h. Regional authorities mostly adopt the national legislation, but specific legislation was created in the north-eastern regions where TBE is endemic. Accordingly, across the country, different monitoring, research, education actions/projects exist, mainly thanks to the initiatives of local health units, research institutions and universities. Most of institutional websites of Italian regions (16 out of 21; Table S1) and *IZS* offer educational and informative materials with key notions about prevention and control of ticks and TBD (e.g., leaflets, online videos and formative courses). Moreover, *IZS* in collaboration with ASL of at least ten regions offer a public service that allows citizens to submit ticks biting humans, for their identification and molecular testing for tick-borne pathogens. 

Notwithstanding, scarce information about human health monitoring and surveillance is published and/or available: only four regional health services annually deliver open-access regional reports about TBD incidence in humans. Some regional health authorities have reported an increase in TBD cases over the years [22,23]. However, due to the difficulties in the diagnostic of these diseases, notifications are scarce. Spotted Fever Group rickettsioses, and Mediterranean Spotted Fever in particular, are considered endemic in Italy, with 1.36 cases of hospitalisation/100,000 person years [24]. Cases of Dermacentor-borne necrosis erythema and lymphadenopathy (DEBONEL/TIBOLA) [25], and of LB [26] are also reported in some regions. However, the most reliable data regard TBE, for which a case definition and more accurate testing exist, according to the 2018 arbovirosis surveillance plan. 

Surveillance on TBD in Italy is mainly a ‘human health’ initiative, focused on human data and expertise. Data and expertise in the animal/environmental domain are only partially considered, although the environmental and animal components (including veterinary health services) are at the basis of the ecological and epidemiological research on TBD. Some TBD, such as anaplasmosis and Q fever, are mainly monitored within the animal health component [27,28,29].

#### 3.1.3. Spain 

**Figure 3:**
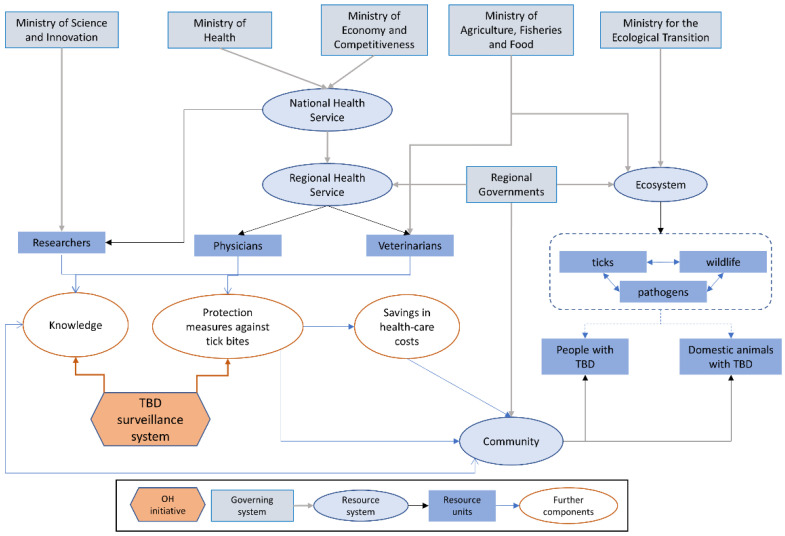
Visual representation of the TBD surveillance initiative in Spain within its system. Relationships (arrows) are classified as governance (grey), membership (black), and causal interactions (blue). The red hexagon represents the initiative with arrows where it impacts the system (modified from Rüegg et al., 2018).

The Ministry of Health together with the Ministry of Agriculture, Fisheries and Food constitute the core of the national health system in terms of human and animal health, respectively. Based on the administrative organisation of the Spanish territory, the health system is structured at three levels (national–regional–local). The above-mentioned ministries are responsible for ensuring the harmonisation of health services, operating at national scale through law-making and its implementation. These institutions, however, devolve governance and decision-making to the regions: 17 autonomous communities plus two Spanish enclaves in North Africa. Thus, regional health services organise and coordinate health services delivery at local level (Figure 3). 

Mediterranean spotted fever (MSF) is endemic across the country and LB and DEBONEL/TIBOLA have been increasingly reported in the last decades [30,31]. Three major human outbreaks of tularemia were identified in 1997, 2007–2009 and 2014–2015 in north-western areas, but only few cases have been associated with tick bites [32,33,34]. Moreover, sporadic cases of tick-paralysis [35], human anaplasmosis [36], babesiosis [37] and tick-borne rickettsioses by *Rickettsia monacensis* have been reported [38]. Since the first description of a patient affected by *Rickettsia sibirica mongolitimonae* infection in 2008, cases and series of adults and children with different clinical manifestations have been published [31,39]. The circulation of CCHF virus was first uncovered in *Hyalomma lusitanicum* ticks collected from red deer of central-western regions in 2010 [40] and, from 2016 to 2021, ten autochthonous human cases of CCHF (three of them fatal) have been notified, some of them retrospectively [41,42,43,44,45]. In addition, the first case of *Neoehrlichia mikurensis* infection in a patient with antecedent of haematological neoplasm has been recently communicated [46], following the first detection of the bacterium in *Ixodes ricinus* ticks removed from cows in the country almost a decade before [47]. 

In Spain, MSF (transmitted by ixodid ticks and caused by *Rickettsia conorii*) and tick-borne relapsing fever (TBRF) (transmitted by soft ticks and caused by *Borrelia recurrentis*) were the only TBD included in the list of notifiable diseases in 1981 (Resolution of the Directorate-General for Public Health, BOE-A-1982-971). In 1996, the National Epidemiological Surveillance Network was created at the service of the National Health System under Royal Decree 2210/1995, 28 December. This modified the system of notification of diseases and adapted it to the requirements of the European Union with the aim of early detection of the population’s health problems and immediate intervention. The autonomous communities in their field of competence developed this regulation and were in charge of periodically sending the established epidemiological information to the Ministry of Health and Consumption. At that time, the list of notifiable diseases included TBE, MSF and TBRF. TBD were considered of regional concern and just a few regions used to report human disease cases (e.g., MSF). In this context, the initiative has mainly evolved focusing on human health and animal health, although research activities at national scale have covered human, animal and environmental domains. Actions concerning the animal domain generally consist of monitoring project-based activities. It is worth noting that Spain (jointly with France, Hungary, Italy and Sweden) participated in a European initiative through the European network for surveillance of TBD (QLK2-CT-2002-01293). As a result of this collaboration, European diagnostic guidelines for tick-borne bacterial diseases were published [48]. Different research groups (the Group of Rickettsiae and Borreliae or the Study Group of Special Pathogens) engaged in the Spanish Society of Infectious Diseases and Clinical Microbiology (SEIMC) [49] and developed multidisciplinary networks, such as the Thematic Network of Cooperative Research of EBATRAG (G03/057), focusing on tick-borne bacterial diseases under laboratorial, clinical and veterinary scope. However, the lack of coordination and resources allocated for TBD determined the disappearance of this study groups. Even so, some Spanish regions (n = 4) count with specific regional TBD monitoring programmes on human and animal health, while the rest (n = 13) are governed by the national legislation (Table S2). For instance, specific programs for emerging research groups of the National Health System (SNS), such as the EMER Program (BOE-A-2008-3288), accredited by the Carlos III Health Institute, have contributed to lead from La Rioja the development of molecular, serological and culture methods and give support to the TBD diagnosis in Spain.

In 2011, the Health Alert and Emergency Coordination Centre (CCAES) from the Ministry of Health, Social Policy and Equality, supported by a group of experts, published the first report on the situation of CCHFV in Spain [50]. In 2015, health authorities defined and implemented new disease surveillance protocols at national scale (Order SSI/445/2015), including TBD that were defined as mandatory communicable diseases (e.g., tularemia) and regional endemic diseases, such as LB. Specific protocols were subsequently designed and rapidly implemented based on necessity, for example, the protocol for surveillance of CCHF released in 2016 [51] based on the updated report on the assessment of the transmission risk of CCHFV [52]. 

Local and/or regional authorities have implemented awareness campaigns through the production of informative materials (e.g., posters, leaflets) and their distribution among health care services and during formative meetings to at-risk categories (e.g., hunters, forest workers, mushroom pickers, etc.). Almost all institutional websites of the regions (15 out of 17; Table S2) offer information related to tick prevention and control. Updated reports about incidence of the most common TBD are also available.

### 3.2. Theory of Change (ToC)

To describe how and why changes are expected to happen with the implementation of surveillance on ticks and TBD, we mapped the theory of change. ToC illustrates the envisioned pathway of change in the underlying system through the inputs, actions and changes in the system and programme (results/outputs) necessary to reach the short- and long-term outcomes, as well as primary and secondary impacts.

**Figure 4:**
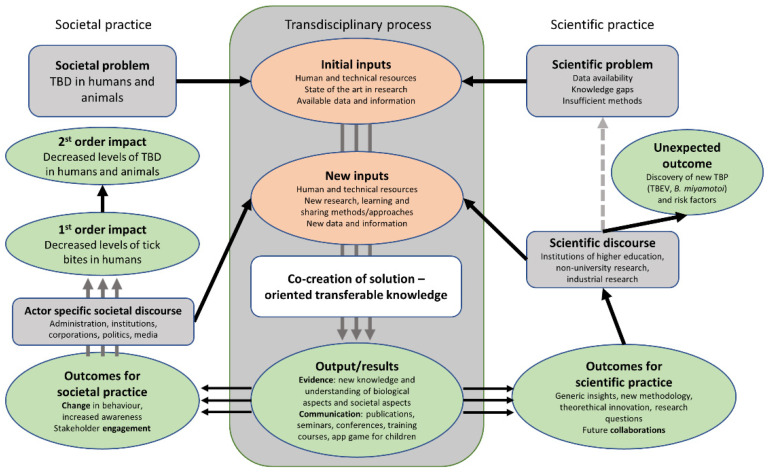
Change pathway for the TBD surveillance system in the Netherlands: inputs from science and society to co-produce outputs taken up by society and the scientific community and disseminated to result in first and second impacts and scientific progress (modified from Rüegg et al., 2018).

Figure 4 describes the pathway of change in The Netherlands. The Dutch initiative has resulted in measurable health benefits, with a reduction in tick bites and LB incidence in humans. In fact, there are chronological differences as the initiative evolves in time, reflected by measurable outcomes. The initiative is focused on ‘event to solution’, and, although it is mainly oriented to damage prevention (“less diseases less expenses”), some activities aim to social changes, such as making landowners responsible for tick bites in their properties. Monitoring and research activities have given rise to increased knowledge by identifying the emergence of new tick-borne pathogens (e.g., *Borrelia miyamotoi* and TBEV), and new risk factors. Actors’ activities and behaviour have been changing with the system evolution; for example, studies are now ongoing to understand if vaccination against TBEV is needed in the country, and a citizen science system to signal exotic tick species (e.g., *Hyalomma* spp.) has been created [53]. 

As regards Italy and Spain, ToC is more difficult to draw because of the lack of data (e.g., assessment of the efficacy of control and surveillance measures) and, as mentioned above, of the variability of actions undertaken in the different administrative regions within each country. 

In Italy, national laws have existed since 1990 to promote diseases notification, but diagnostic difficulties probably hinder notifications and regional data are not displayed/made public on a national platform. Regional health services are more or less active, depending on the epidemiological situation of the region. The same happens for the research at regional level: despite the limited resources, the research activities have contributed to gain knowledge on the distribution of tick vectors and transmitted pathogens in the country, with the discovery of new pathogens, e.g., *Borrelia miyamotoi* and ‘*Candidatus* Rickettsia rioja’ [54,55] and eco-epidemiological determinants of disease (e.g., ticks expanding their geographic range [56]). Moreover, new legislation on TBE has been issued, following European legislation (Ministry of Health Circular No. 17500, 2018).

In Spain, the initiative has evolved through application and adaptation of surveillance protocols, but also extending the surveillance to the entire national territory. The initiative demonstrated flexibility to change over time and according to needs; for example, new protocols and activities were created following the discovery of CCHFV. As another example, according to the laboratory environmental findings (surveillance of risk through the study of ticks), efforts have been made to draw haematologists and oncologists’ attention to the importance of certain TBD (e.g., neoehrlichiosis) in patients under these conditions in tick-endemic areas. Thus, sessions in scientific conferences have been held and research projects offering the possibility of incorporating patients under diagnostic suspicion within specialised clinical networks (e.g., Spanish Network of Infectious Pathology, REIPI) have been developed. At the educational level, in the last years, workshops, talks or round tables focused on TBD under a OH approach have been organised to give advice on the prevention of TBD to schoolchildren, university students and the elderly, among others, during the celebration of the Science Week or the International Day of Women and Girls in Science.

### 3.3. The Degree of One Health 

The level of OH implementation was individually evaluated for the three surveillance systems under study. As already pointed out, in Spain and Italy we were only able to perform a partial semi-quantitative evaluation of these health initiatives. OH sharing was evaluated qualitatively and systemic organisation could not be assessed due to the complexity and fragmentation of the systems (Table 1).

#### 3.3.1. The Netherlands

Mean scores of OH aspects suggest a good balance between operations and infrastructures of the surveillance system in place, as it can be observed in the spider diagram in Supplementary File S1. The Dutch health initiative obtained a perfect match in OH planning, OH working, OH sharing and Systemic organisation (mean score: 1.0). High mean scores were also obtained for OH thinking and OH learning (0.90 and 0.80, respectively). 

Regarding OH thinking, different dimensions and scales were identified as being relevant elements within the initiative. Management and legislation have a passive recognition since the tick surveillance system is part of the national programme for the control of zoonoses. This programme has an impact both at local (e.g., province and/or regions) and national level and foresees a high level of transdisciplinary and trans-sectoral collaboration between public, animal, and environmental health. Nevertheless, TBD are not seen as a ‘veterinary’ concern, so data and expertise in the animal domain are only partially considered (e.g., scarce information on TBD in domestic animals is available). The initiative considers several dimensions of life, from genes (research on microorganisms) to individuals, to the population scale. Time dimension is of great relevance especially when it comes to the surveillance program design and implementation (e.g., organisation and funding); in fact, like other vector-borne diseases, TBD incidence is seasonal, concurring with the activity period of ticks (e.g., early spring to autumn in the case of *I. ricinus*). Time is also a risk factor for the health issue, considering the time-lapse between the human/animal exposure to infected ticks, pathogen transmission after a tick bite and the onset of disease symptoms. Social and economic resources are relevant and well-considered within the health system, and economic and societal costs of some TBD have been assessed. The Dutch initiative is economically supported by the Dutch Ministry of Health, and, to a lesser extent, by international institutions. 

One Health learning reflects the good cooperation among the stakeholders involved at different levels in the initiative. Informative meetings occur regularly, leading to the sharing and discussion of information and perspectives, aimed at informing decision makers and the general population. 

With the OH planning assessment, we identified that responsibilities and professional skills are highly supportive of a OH approach. Common aims focused on transdisciplinarity were detected, as was also observed at the organisational level. The actors involved in the initiative are well identified and their activity is supported by the active participation of stakeholders (e.g., citizens involved in tick bites reporting, participation to questionnaires, use of the mobile apps, etc.). Though adequate economic resources underpin most of the actions, critical aspects concern the inability for active participation to the initiative of some stakeholders (e.g., landowners) and the economic sustainability in the long-term of some monitoring activities. 

As regards transdisciplinarity, the Dutch initiative is broad and inter-sectoral, with great participation of the non-scientific community. Strong engagement of some categories of scientists was highlighted (e.g., medical doctors, environmental specialists); as mentioned above, veterinary professionals/practitioners are only partially involved. There is generally a good interaction among actors within the initiative, fostering the collaboration with spontaneous and frequent in-person meetings or staff-exchange. The empowerment of the initiative is also reflected across different social classes, as it was revealed from social studies performed among the general population [57,58] or those addressed to groups at risk [59,60,61]. 

The initiative is endowed with internal and external mechanisms for information exchange among stakeholders, including the general population. Appropriate institutional databases and backup systems ensure data storage. However, the sharing of the methodologies applied and results obtained is compartmentalised, and communication is mainly linked to specific projects. For instance, the National Institute for the Public Health and the Environment (RIVM) coordinates and organises annual meetings and workshops concerning LB projects, during which all national actors/stakeholders can meet and interact. External sharing mechanisms comprise the participation in international congresses/workshops alongside article publication on international scientific journals. Moreover, RIVM accounts with an institutional website where it regularly publishes open-access reports about monitoring and surveillance activities. 

#### 3.3.2. Italy

The OH thinking of the Italian initiative had an average score of 0.73. Lower values were assigned to OH learning (0.45), OH planning (0.44) and OH working (0.41) (Table 1; Supplementary File S2). The initiative extends at national level but is implemented at regional level, and is mostly addressed to containing and/or preventing damage, especially in humans. System fragmentation mirrors notable differences among regions, related to the variable epidemiological situation and the availability of resources for TBD surveillance. 

Legislation on TBD ranges from regional law to national regulations. Notwithstanding, law enforcement is rather fragmented, with TBE being the disease that receives higher attention. 

All dimensions of life involved in the health issue are fully considered (i.e., from pathogens to tick vectors, vertebrate hosts populations and the environment), as well as the time dimension (as described above for the Netherlands). Socio-health and economics dimensions are only partly considered (e.g., free/discounted TBEV immunisation are offered to citizens in endemic areas). Most monitoring and risk assessment activities on TBD are short-term and mainly based on research funds; thus, limited resources lead to slight support for surveillance actions. Some health strategies and allocated resources are not specifically addressed to TBD: for instance, TBE surveillance is included within the national surveillance programme for arbovirosis, in which activities and resources mainly address mosquitoes-transmitted diseases. Notwithstanding, supportive resources are partially allocated for the reduction in incidence of tick bites and TBE in humans, through information campaigns and vaccination programmes. 

Veterinary and human medicine are the main disciplines involved in TBD surveillance. Ecologists, data analysts and the non-scientific community are marginally considered. According to Davis et al. [62], the Italian initiative can be classified as a ‘One Medicine’ initiative rather than ‘One Health’. The veterinary sector seems to be more engaged than the public health sector: research products generated by veterinarians and/or biologist are more numerous than those related to the human medical sector. Stakeholders’ involvement is limited to regional and local initiatives. The non-profit association ‘Lyme Italia’ [63], for example, collaborates with some regions by organising information campaigns, congresses and informative meetings to increase awareness about TBD. 

Regarding OH learning, it can be pointed out that some health institutions, such as Experimental Zooprophylactic Institutes (*IZS*), have contributed to basic learning through educational online courses, especially addressed to medical doctors and veterinarians. Stakeholders’ involvement and awareness has also supported the adaptive learning of the health system (e.g., recognition of LB as a ‘rare disease’ in some regions or professional categories, with economic support to the patients). 

Knowledge sharing and data sharing was difficult to assess in the Italian context because of system fragmentation. We had difficulties finding available and/or updated information on TBD incidence at national level. Information was also sparsely available at local level and it was updated only in some of the regions (e.g., Autonomous Province of Trento and Bolzano, Veneto and Piedmont regions; Table S1). Data flows among actors is compartmentalised, with information exchange limited amongst research groups. Notwithstanding, research studies are shared with the scientific community through publications in national and international journals and the participation to regional, national and international congresses. Accordingly, the fragmentation of the system made it impossible to assess teamwork, leadership and governance characteristics within the systemic organisation.

#### 3.3.3. Spain

The OH evaluation of initiative implemented in Spain had similar results to the Italian initiative. Better results were obtained for OH thinking (mean score: 0.78), and lower indexes for OH learning (0.6), OH working (0.51), OH planning (0.49) (Table 1; Supplementary File S3).

The same dimensions described for health initiatives of the Netherlands and Italy were considered for Spain. The geographic dimension is of great relevance, since the health issue extends at country level, including different ecological regions. Several levels of dimension of life were considered, from genes to cells, tick vectors, domesticated and wild animals, humans and populations. Legislation is also significant: most TBD are regulated by law and subjected to mandatory surveillance and notification. Legislation supplies surveillance protocols for guidance to health care services. Alongside surveillance activities, regulations on TBD have evolved over the years by being enforced from regional to national scales. Notwithstanding, flexibility and/or adaptation are allowed according to the epidemiological situation. 

The surveillance initiative focuses on preventing damage; it is not carried out as a unique strategy, but it is composed by several separate grant-based projects. Social and economic factors are fully considered, especially for LB (e.g., clinical cases with long-term unspecific symptoms) and CCHF (due to the possible deadly consequences). Moreover, professional at-risk categories have been object of research studies for a long time and are the main target for awareness campaigns. Accordingly, TBD are considered as ‘occupational diseases’ by the Spanish law (Royal Decree 664/1997).

The Spanish health system has demonstrated a great response capacity and adaptation over the years. Thanks to the presence of dynamic feedback loops the system has been able to adapt to health necessities. Indeed, TBD surveillance was promoted from regional to national level by establishing standardised monitoring protocols (e.g., for MSF in 2013). Moreover, in the light of the first human cases of CCHF in 2016, the health system moved rapidly towards emergency control [64]. This emerging problem caused the system to rely on the union of effort and synergies for carrying out strategic risk assessments at country level [51] and specific protocols for disease surveillance in humans were implemented. 

Regarding OH planning and OH working, the initiative can be regarded as a ‘One Medicine’ approach, skewed towards human medicine. However, in some local/regional contexts, the activity of veterinary sector predominates over human medicine. There is a reasonable identification of the stakeholders involved in the initiative and punctual collaboration between human and veterinary disciplines. Over the years, national health institutions, in coordination with regional health services and reference hospitals and laboratories, have worked together for disease surveillance and control in humans. New research groups were established, but time and resources were not well allocated to sustain the activity long-term, leading to their disappearance. Nowadays, collaborations are in place among research groups, but their activity is mainly based on grants. The non-scientific community is marginally involved, with short-term collaborations with representatives of agricultural sector, hunting associations, bird ringers or workers collaborating in projects with endangered animal species. 

Although the health system is endowed with good channels of communication, the information and knowledge generated within the Spanish initiative is somehow compartmentalised and mainly limited to scientists. Notwithstanding, data sharing is complemented by the production of national and/or regional annual reports (Table S2), the publication of scientific articles and the participation to national and international scientific events. The knowledge gained within the experience is not only collected and stored but it is partially shared and discussed among actors, leading to changes on the basis and objectives; however, it depends on economic resources, considering that most studies performed are grant-based projects. As in the case of Italy, systemic organisation could not be assessed because of the fragmentation of the system.

## 4. Discussion

The purpose of our research was to describe the surveillance initiatives on TBD in the Netherlands, Italy and Spain to assess their degree of OH implementation and highlight strengths and weaknesses, considering the intrinsic differences of the three systems. The idea was not to define which is the best system, but to identify supposedly ideal elements for disease monitoring and prevention that can be applied in different contexts. Administrative structure, health legislation, ecology and epidemiology of diseases condition the surveillance activities in place in each country, though the same European regulations are followed. Even if the harmonisation of activities is difficult, countries can learn from each other to improve their systems with a OH-oriented approach.

The evaluation protocol developed by NEOH was useful to analyse in detail the surveillance initiatives. Unfortunately, we had difficulties in compiling some aspects related to Italy and Spain; in fact, surveillance on TBD in these countries is composed by several local initiatives, due to legislation and to the specific epidemiological situation of the different regions within these countries. 

The One Health assessment enabled to appreciate the presence of a multidisciplinary approach and cross-sectoral collaboration, especially in the Netherlands. The Dutch monitoring, surveillance and research activities extend to the national level and are based on the collaboration between different disciplines related to public, animal and environmental health. For the health initiatives implemented in Italy and Spain, the evaluation revealed that the underlying mechanisms are mainly based on a ‘One Medicine’ approach, skewed to the human sector. Notwithstanding, punctual collaborations between several scientific disciplines and non-scientific communities have been in place over time in some administrative regions/provinces. Despite the human health dominant approach in the Spanish and Italian health systems, the veterinary component seemed to contribute most to scientific knowledge in these initiatives. Differences among countries may be due to the different epidemiological situations and health impacts of ticks/TBD on the human health and animal health sectors. In the Netherlands, like other countries of northern EU, ticks seem of minor concern for the animal production sector, also due to misdiagnoses and a low level of livestock owners’ awareness [65]; conversely, cases of LB in humans have been notified since at least 1994 [66]. In southern European countries, in contrast, tick genera such as *Rhipicephalus* and *Hyalomma*, transmit anaplasmosis and babesiosis, which can impact livestock health and production [67,68,69]. In fact, in Italy, TBD are mostly studied by zooprophylactic diagnostic institutes, which are animal health institutions. In Spain and Italy, TBD are considered emerging threats for human health and the human medicine interest for these diseases has been growing only recently.

Monitoring of tick vectors should be at the basis of any TBD surveillance system. The use of proper methods and sampling strategies may contribute to the preparedness of health systems, with solid knowledge about the epidemiological situation in a given area [70]. Regarding human health, TBD surveillance across EU-member states is rather heterogeneous [71], even if Lyme neuroborreliosis, TBE and CCHF are subjected to mandatory reporting. Legislation, however, does not ensure the effectiveness for disease monitoring. For instance, TBD reporting in the Netherlands is subjected to passive surveillance and disease-case reporting is voluntary. Notwithstanding, medical doctors routinely report tick bites and disease cases and thereby, contribute to the general picture of LB at country level [72]. By contrast, Italian legislation requires mandatory reporting of zoonotic TBD, but reliable data are available mainly on TBE, and the burden of LB is uncertain. The same uncertainty on the number of LB cases occurs in Spain, recent statistics reveal that the number of LB has tripled in the last 15 years, showing an increase in hospitalisations of more than 191% [73]. However, these data are not accurate at all and must be interpreted with caution. It is true that now the disease is better known, the number of human tick bites has been increasing in the last years, wildlife is more protected and subsequently, there are more amplifiers/reservoirs to complete ticks’ life cycle, the livestock production is globally expanding and predators of ticks (ants, wasps…) are decreasing as a consequence of the use of pesticides [74,75]. It must, however, be recognised that there is a lot of over- and disinformation and greater awareness by the population because the problem appears even in popular magazines (it is in the news that celebrities such as Alec Baldwin, Richard Gere or Justin Bieber suffer from LB). In addition, many scientific data are based on seroprevalence studies, although serology is not a good tool to distinguish between active or past infection (even worse in tick-endemic areas) [76]. The reality is that those patients (having the same problem now and before) were not previously ‘labelled’ as diagnosed with LB but with other pathologies such as radiculopathy, for instance.

Contrasting scenarios among countries may be explained by differences in the structural and operational aspects discerned during the evaluation process. Surveillance characteristics might also be a result of the experience gained by the three countries on zoonotic disease surveillance. In the Netherlands, the outbreaks of avian influenza in 2003 and Q fever in 2007 brought to the creation of a consolidated intersectoral network for the surveillance, monitoring and signalling of zoonotic diseases [77]. Such structure also served as a basis for surveillance of TBD. Ongoing climate and environmental changes, favouring a higher risk of some TBD, could increase the awareness on the importance of intersectoral surveillance networks also in Italy and Spain. 

Stakeholders have demonstrated strongly supporting the health initiatives when they are involved, by contributing to increase knowledge and helping to achieve main goals. For instance, in the Dutch initiative, citizens actively participated in several projects helping to monitor tick bites and LB across the national territory [57,78]. Moreover, their contribution has enabled the Dutch initiative for reflexion and self-assessment, but also for measuring its impacts on health [10,11,60,79,80]. To maintain public engagement, it is of paramount importance to give information feedbacks related to the activities in which stakeholders are involved [12]. Efficient information exchange among actors and stakeholders leads to health benefits by a prompt system reaction (response capacity) and control of the health issue [81]. Information exchange was generally compartmentalised for all three initiatives evaluated, but greater sharing was observed for the Netherlands. However, also the Spanish health system demonstrated efficiency and preparedness with the early detection of CCHF [64]. 

Dedicated time and availability of economic resources are among the major limiting factors for the maintenance of health initiatives. This was observed in Italy and Spain in particular, where resources are mainly allocated for TBE and CCHF, respectively. This approach, even if reasonable since these are the main health issues, does limit the effectiveness of control strategies for other TBD and may reduce the societal impact of the initiative. 

Some of the aspects discussed above were included in the recommendations proposed by the ECDC to tackle TBD [64]. Amongst recommendations, the assessment of the effects of surveillance initiatives is of paramount importance. Indeed, regular evaluation is needed to ensure the effectiveness of the surveillance, its efficiency and operation. Different aspects can be evaluated [82]. By using the NEOH framework, we specifically focused on the evaluation of the surveillance integration and on the degree of OH implementation within the three surveillance systems. We observed that intersectoral collaboration and communication are key elements of an effective surveillance; good practices that foster such interdisciplinarity are more and more important to tackle complex health issues, such as vector-borne diseases.

## 5. Conclusions

Surveillance systems characterised by transdisciplinary collaborations might be more effective in disease prevention and early response to emerging health threats, including tick-borne diseases. The semi-quantitative NEOH evaluation protocol is a useful tool to highlight the strengths and weaknesses of integrated surveillance, and help to inform decision makers about the importance of adopting an integrated approach for zoonosis surveillance and management.

## Figures and Tables

**Table 1:** Scores attributed to operational and infrastructural dimensions of the surveillance initiatives.

Country	Thinking	Planning	Working	Learning	Sharing	Systemic Organisation
the Netherlands	0.9	1	1	0.8	1	1
Italy ^1^	0.73	0.44	0.40	0.45	na	na
Spain ^1^	0.78	0.49	0.51	0.6	na	na

^1^ na = not assessed: sharing and systemic organisation were not quantitatively evaluated for Italy and Spain due to the administrative fragmentation of the initiatives in these two countries.

## Data Availability

The datasets compiled in this study can be found in the Supplementary Materials.

## Supplementary Materials

The following supporting information can be downloaded at: www.mdpi.com/article/10.3390/vetsci9090504/s1, Table S1: Description of surveillance and monitoring activities on TBD across Italian regions; Table S2: Description of surveillance and monitoring activities on TBD across Spanish regions; Supplementary File S1: Evaluation of the degree of OH implementation of the Dutch surveillance system on TBD, based on the NEOH template; Supplementary File S2: Evaluation the degree of OH implementation of the Italian surveillance system on TBD, based on the NEOH template; Supplementary File S3: Evaluation the degree of OH implementation of the Spanish surveillance system on TBD, based on the NEOH template.

## References

[1] Medlock J.M., Hansford K.M., Bormane A., Derdakova M., Estrada-Peña A., George J.C., Golovljova I., Jaenson T.G., Jensen J.K., Jensen P.M. (2013). Driving forces for changes in geographical distribution of Ixodes ricinus ticks in Europe. Parasites Vectors.

[2] European Food Safety Authority, EFSA. EFSA and ECDC Join Forces to Fight Vector-Borne Diseases, 2014. https://www.efsa.europa.eu/en/press/news/efsa-and-ecdc-join-forces-fight-vector-borne-diseases.

[3] van den Wijngaard C.C., Hofhuis A., Simões M., Rood E., van Pelt W., Zeller H., Van Bortel W. (2017). Surveillance perspective on Lyme borreliosis across the European Union and European Economic Area. Euro Surveill..

[4] Clow K.M., Leighton P.A., Pearl D.L., Jardine C.M. (2019). A framework for adaptive surveillance of emerging tick-borne zoonoses. One Health.

[5] Network for Evaluation of One Health (NEOH). https://neoh.onehealthglobal.net/.

[6] Rüegg S.R., Nielsen L.R., Buttigieg S.C., Santa M., Aragrande M., Canali M., Ehlinger T., Chantziaras I., Boriani E., Radeski M. (2018). A Systems approach to evaluate One Health initiatives. Front. Vet. Sci.

[7] Rüegg S.R., Häsler B., Zinsstag J. (2018). Integrated Approaches to Health: A Handbook for the Evaluation of One Health.

[8] van den Wijngaard C.C., Hofhuis A., Wong A., Harms M.G., de Wit G.A., Lugnér A.K., Suijkerbuijk A.W.M., Mangen M.J.J., van Pelt W. (2017). The cost of Lyme borreliosis. Eur. J. Public Health.

[9] National Institute for the Public Health and the Environment, RIVM Spread of Tick-Borne Encephalitis Virus in the Netherlands, 2020. https://www.rivm.nl/en/news/spread-of-tick-borne-encephalitis-virus-in-netherlands.

[10] van den Wijngaard C.C., Brown V., Harms M.G., van Pelt W., Hofhuis A. Increase in the incidence and burden of Lyme borreliosis in the Netherlands between 2014 and 2017. National Institute for Public Health and the Environment, RIVM, 2017. https://www.rivm.nl/sites/default/files/2019-04/19.%20Hofhuis_Lyme%20borreliosis.pdf.

[11] Hofhuis A., Bennema S., Harms M., van Vliet A.J., Takken W., van den Wijngaard C.C., van Pelt W. (2016). Decrease in tick bite consultations and stabilization of early Lyme borreliosis in the Netherlands in 2014 after 15 years of continuous increase. BMC Public Health.

[12] European Centre for Disease Prevention and Control, ECDC Synergies in Community and Institutional Public Health Emergency Preparedness for Tick-Borne Diseases in the Netherlands. A Case Study on Tick-Borne Encephalitis and Lyme Borreliosis. Stockholm, 2018. https://www.ecdc.europa.eu/sites/default/files/documents/Lyme-TBE-Netherlands-emergency-preparedness-country-visit-report.pdf.

[13] Jahfari S., de Vries A., Rijks J.M., Van Gucht S., Vennema H., Sprong H., Rockx B. (2017). Tick-Borne encephalitis virus in ticks and roe deer, the Netherlands. Emerg. Infect. Dis..

[14] De Graaf J.A., Reimerink J.H., Voorn G.P., Bij de Vaate E.A., de Vries A., Rockx B., Schuitemaker A., Hira V. (2016). First human case of tick-borne encephalitis virus infection acquired in the Netherlands, July 2016. Euro Surveill..

[15] Weststrate A.C., Knapen D., Laverman G.D., Schot B., Prick J.J., Spit S.A., Reimerink J., Rockx B., Geeraedts F. (2017). Increasing evidence of tick-borne encephalitis (TBE) virus transmission, the Netherlands, June 2016. Euro Surveill..

[16] Esser H.J., Liefting Y., Ibáñez-Justicia A., van der Jeugd H., van Turnhout C.A.M., Stroo A., Reusken C.B.E.M., Koopmans M.P.G., de Boer W.F. (2020). Spatial risk analysis for the introduction and circulation of six arboviruses in the Netherlands. Parasites Vectors.

[17] Uiterwijk M., Ibáñez-Justicia A., van de Vossenberg B., Jacobs F., Overgaauw P., Nijsse R., Dabekaussen C., Stroo A., Sprong H. (2021). Imported *Hyalomma* ticks in the Netherlands 2018–2020. Parasites Vectors.

[18] National Institute for Public Health and the Environment, RIVM. https://www.rivm.nl/.

[19] STIGAS Week van de teek, 2022. https://www.weekvandeteek.nl/.

[20] National Institute for Public Health and the Environment, RIVM Educational Video on Tick Bites and Lyme Borreliosis, 2012. https://www.youtube.com/watch?v=MveB_UYn8cY&list=UUVHvRJWSXP_txjODgD61sKg&index=2.

[21] Tekenradar. https://www.tekenradar.nl/.

[22] Regional Service for the Epidemiology of Infectious Diseases, SeREMI Zoonotic Diseases, 2019. https://www.seremi.it/viz-condizioni/Malattie%20zoonotiche/.

[23] Alfano N., Tagliapietra V., Rosso F., Ziegler U., Arnoldi D., Rizzoli A. (2020). Tick-borne encephalitis foci in northeast Italy revealed by combined virus detection in ticks, serosurvey on goats and human cases. Emerg. Microbes. Infect..

[24] Gomez-Barroso D., Vescio M.F., Bella A., Ciervo A., Busani L., Rizzo C., Rezza G., Pezzotti P. (2019). Mediterranean spotted fever rickettsiosis in Italy, 2001-2015: Spatio-temporal distribution based on hospitalization records. Ticks Tick Borne Dis..

[25] Selmi M., Bertolotti L., Tomassone L., Mannelli A. (2008). *Rickettsia slovaca* in *Dermacentor marginatus* and Tick-borne lymphadenopathy, Tuscany, Italy. Emerg. Infect. Dis..

[26] Stroffolini G., Segala F.V., Lupia T., Faraoni S., Rossi L., Tomassone L., Zanet S., De Rosa F.G., Di Perri G., Calcagno A. (2021). Serology for *Borrelia* spp. in Northwest Italy: A Climate-matched 10-Year Trend. Life.

[27] Alberti A., Zobba R., Chessa B., Addis M.F., Sparagano O., Pinna Parpaglia M.L., Cubeddu T., Pintori G., Pittau M. (2005). Equine and canine *Anaplasma phagocytophilum* strains isolated on the island of Sardinia (Italy) are phylogenetically related to pathogenic strains from the United States. Appl. Environ. Microbiol..

[28] Zobba R., Anfossi A.G., Pinna Parpaglia L., Dore G.M., Chessa B., Spezzigu A., Rocca S., Visco S., Pittau M., Alberti A. (2014). Molecular investigation phylogeny of *Anaplasma* spp. in Mediterranean ruminants reveal the presence of neutrophil-tropic strains closely related to *A. platys*. Appl. Environ. Microbiol..

[29] Barlozzari G., Sala M., Iacoponi F., Volpi C., Polinori N., Rombolà P., Vairo F., Macrì G., Scarpulla M. (2020). Cross-sectional serosurvey of *Coxiella burnetii* in healthy cattle and sheep from extensive grazing system in central Italy. Epidemiol. Infect..

[30] Romaní Vidal A., Fernández-Martínez B., Herrador Z., León Gómez I., Gómez Barroso D. (2020). Spatial and temporal trends of Mediterranean spotted fever in Spain, 2005–2015. Ticks Tick Borne Dis..

[31] Portillo A., Santibáñez S., García-Álvarez L., Palomar A.M., Oteo J.A. (2015). Rickettsioses in Europe. Microbes. Infect..

[32] De Mateo S., Ruiz Coisin C. (1998). Outbreak of tularaemia in Castilla y Leon, Spain. Euro Surveill..

[33] Allue M., Sopeña C.R., Gallardo M.T., Mateos L., Vian E., Garcia M.J., Ramos J., Berjon A.C., Viña M.C., Garcia M.P. (2008). Tularemia outbreak in Castilla y Leon, Spain, 2007: An update. Euro Surveill..

[34] Mínguez-González O., Gutiérrez-Martín C.B., Martínez-Nistal M.D.C., Esquivel-García M.D.R., Gómez-Campillo J.I., Collazos-Martínez J.Á., Fernández-Calle L.M., Ruiz-Sopeña C., Tamames-Gómez S., Martínez-Martínez S. (2021). Tularemia outbreaks in Spain from 2007 to 2020 in humans and domestic and wild animals. Pathogens.

[35] Oteo J.A., Maraví E., Martínez de Artola V., Antuñano P. (1990). Parálisis por mordedura de garrapata. Med. Clin..

[36] García J.C., Núñez M.J., Portillo A., Oteo J.A. (2015). Human anaplasmosis: Two case-reports. Enferm. Infecc. Microbiol. Clin..

[37] Gonzalez L.M., Rojo S., Gonzalez-Camacho F., Luque D., Lobo C.A., Montero E. (2014). Severe babesiosis in immunocompetent man, Spain, 2011. Emerg. Infect. Dis..

[38] Jado I., Oteo J.A., Aldámiz M., Gil H., Escudero R., Ibarra V., Portu J., Portillo A., Lezaun M.J., García-Amil C. (2007). *Rickettsia monacensis* and human disease, Spain. Emerg. Infect. Dis..

[39] Santibáñez S., Ramos J.M., Sanjoaquín I., Guillén S., Llorente M., Lozano M.D.C., Ramírez de Arellano E., Cervera-Acedo C., García-García C., Santibáñez P. *Rickettsia sibirica mongolitimonae* infection. The CRETAV experience (Poster number: 0763). Proceedings of the XXV Conference of the Spanish Society of Infectious Diseases and Clinical Microbiology.

[40] Estrada-Peña A., Palomar A.M., Santibáñez P., Sánchez N., Habela M.A., Portillo A., Romero L., Oteo J.A. (2012). Crimean-Congo hemorrhagic fever virus in ticks, Southwestern Europe, 2010. Emerg. Infect. Dis..

[41] National Epidemiological Surveillance Network, RENAVE Annual Report about Communicable Disease, Period 2017–2018. https://www.isciii.es/QueHacemos/Servicios/VigilanciaSaludPublicaRENAVE/EnfermedadesTransmisibles/Documents/INFORMES/INFORMES%20RENAVE/RENAVE_Informe_anual__2017-2018.pdf.

[42] Coordination Centre for Health Alerts and Emergencies, CCAES Rapid Risk Assessment on Crimean-Congo Haemorrhagic Fever in Salamanca Province, 2020. https://www.mscbs.gob.es/en/profesionales/saludPublica/ccayes/alertasActual/Crimea_Congo/docs/20200827_ERR_Crimea_Congo_Salamanca.pdf.

[43] European Centre for Disease Prevention and Control, ECDC Communicable Diseases Report, 2021. https://www.ecdc.europa.eu/sites/default/files/documents/Communicable-disease-threats-report-1-may-2021-allusers.pdf.

[44] National Epidemiological Surveillance Network, RENAVE Crimean-Congo Haemorrhagic Fever. Cases Notified to RENAVE, 2021. https://www.isciii.es/QueHacemos/Servicios/VigilanciaSaludPublicaRENAVE/EnfermedadesTransmisibles/Documents/archivos%20A-Z/Fiebre_Hemorr%C3%A1gica_Crimea_Congo/Informe%20final_casos%20FHCC_RENAVE_2021.pdf.

[45] Negredo A., Sánchez-Ledesma M., Llorente F., Pérez-Olmeda M., Belhassen-García M., González-Calle D., Sánchez-Seco M.P., Jiménez-Clavero M.Á. (2021). Retrospective identification of early autochthonous case of Crimean-Congo hemorrhagic fever, Spain, 2013. Emerg. Infect. Dis..

[46] González-Carmona P., Portillo A., Cervera-Acedo C., Vargas-Pabón M., Muñiz-Lobato S., García-Iglesias L., Blanco-Costa M.I., González-Fernández D., Ramiro-Bejarano I.M., Ferreras-García A. First confirmed case of ‘*Candidatus* Neoehrlichia mikurensis’ infection in a patient with antecedent of hematological neoplasm in Spain. Proceedings of the International Intracellular Bacteria Meeting 2022.

[47] Palomar A.M., García-Álvarez L., Santibáñez S., Portillo A., Oteo J.A. (2014). Detection of tick-borne *’Candidatus* Neoehrlichia mikurensis’ and *Anaplasma phagocytophilum* in Spain in 2013. Parasites Vectors.

[48] Brouqui P., Bacellar F., Baranton G., Birtles R.J., Bjoërsdorff A., Blanco J.R., Caruso G., Cinco M., Fournier P.E., Francavilla E. (2004). Guidelines for the diagnosis of tick-borne bacterial diseases in Europe. Clin. Microbiol. Infect..

[49] Spanish Society of Infectious Diseases and Clinical Microbiology (SEIMC). https://seimc.org/.

[50] Suárez B., Sierra M.J., Cortés M., Jansa J.M., Romero L.J., Estrada-Peña A., Tenorio A., Negredo A.I., Fernández M.D., Sánchez L.P. Situation Report and Risk Assessment Transmission of Crimean-Congo Hemorrhagic Fever (CCHF) in Spain; Health Alert and Emergency Coordination Center (CCAES) from the Ministry of Health, Social Policy and Equality. General Directorate for Public Health and Foreign Health; Government of Spain: Madrid, Spain, 2011. https://www.sanidad.gob.es/profesionales/saludPublica/ccayes/alertasActual/Crimea_Congo/docs/ACTUALIZACION_ER_FHCC_20160916.pdf.

[51] Coordination Centre for Health Alerts and Emergencies, CCAES Protocol for Surveillance of Crimean Congo Haemorrhagic Fever, CCHF, 2017. https://www.mscbs.gob.es/profesionales/saludPublica/ccayes/alertasActual/Crimea_Congo/docs/16.06.2017-Protocolo-vigilancia-FHCC.pdf.

[52] Suárez B., Sierra M.J., García San Miguel L., Palmera R., Reques L., Montero L., Simón F., Romero L.J., Estrada-Peña A., Sánchez-Seco M.P. (2016). Situation Report and Assessment of the Transmission Risk of Crimean-Congo Haemorrhagic Fever (CCHF) in Spain.

[53] Netherlands Food and Consumer Product Safety Authority, NVWA, 2022. https://www.nvwa.nl/onderwerpen/muggen-knutten-en-teken/teken.

[54] Ravagnan S., Tomassone L., Montarsi F., Krawczyk A.I., Mastrorilli E., Sprong H., Milani A., Rossi L., Capelli G. (2018). First detection of *Borrelia miyamotoi* in *Ixodes ricinus* ticks from northern Italy. Parasites Vectors.

[55] Garcia-Vozmediano A., Giglio G., Ramassa E., Nobili F., Rossi L., Tomassone L. (2020). *Dermacentor marginatus* and *Dermacentor reticulatus*, and their infection by SFG rickettsiae and *Francisella*-like endosymbionts, in mountain and periurban habitats of northwestern Italy. Vet. Sci..

[56] Garcia-Vozmediano A., Krawczyk A.I., Sprong H., Rossi L., Ramassa E., Tomassone L. (2020). Ticks climb the mountains: Ixodid tick infestation and infection by tick-borne pathogens in Western Alps. Ticks Tick Borne Dis..

[57] Mulder S., van Vliet A.J., Bron W.A., Gassner F., Takken W. (2013). High risk of tick bites in Dutch gardens. Vector Borne Zoonotic Dis..

[58] van der Heijden A., Mulder B.C., Poortvliet P.M., van Vliet A.J.H. (2017). Social-cognitive determinants of the tick check: A cross-sectional study on self-protective behavior in combatting Lyme disease. BMC Public Health.

[59] Moll van Charante A.W., Groen J., Osterhaus A.D. (1994). Risk of infections transmitted by arthropods and rodents in forestry workers. Eur. J. Epidemiol..

[60] Beaujean D.J., Gassner F., Wong A., Steenbergen J.E., Crutzen R., Ruwaard D. (2016). Education on tick bite and Lyme borreliosis prevention, aimed at schoolchildren in the Netherlands: Comparing the effects of an online educational video game versus a leaflet or no intervention. BMC Public Health.

[61] Jahfari S., Herremans T., Platonov A.E., Kuiper H., Karan L.S., Vasilieva O., Koopmans M.P., Hovius J.W., Sprong H. (2014). High seroprevalence of *Borrelia miyamotoi* antibodies in forestry workers and individuals suspected of human granulocytic anaplasmosis in the Netherlands. New Microbes New Infect..

[62] Davis M.F., Rankin S.C., Schurer J.M., Cole S., Conti L., Rabinowitz P. (2017). COHERE Expert Review Group. Checklist for One Health Epidemiological Reporting of Evidence (COHERE). One Health.

[63] Associazione Lyme Italia e coinfezioni. https://www.associazionelymeitalia.org/.

[64] European Centre for Disease Prevention and Control Synergies in Community and Institutional Public Health Emergency Preparedness for Tick-Borne Diseases in Spain and the Netherlands. Stockholm, 2018. https://www.ecdc.europa.eu/en/publications-data/synergies-community-and-institutional-public-health-emergency-preparedness-tick-1.

[65] Johansson M., Mysterud A., Flykt A. (2020). Livestock owners’ worry and fear of tick-borne diseases. Parasites Vectors.

[66] Hofhuis A., Harms M., van den Wijngaard C., Sprong H., van Pelt W. (2015). Continuing increase of tick bites and Lyme disease between 1994 and 2009. Ticks Tick Borne Dis..

[67] Torina A., Caracappa S. (2007). Anaplasmosis in cattle in Italy. Vet. Res. Commun..

[68] Silaghi C., Nieder M., Sauter-Louis C., Knubben-Schweizer G., Pfister K., Pfeffer M. (2018). Epidemiology, genetic variants and clinical course of natural infections with *Anaplasma phagocytophilum* in a dairy cattle herd. Parasites Vectors.

[69] Moraga Fernández A., Ortiz J.A., Jabbar A., Ghafar A., Cabezas-Cruz A., de la Fuente G., de la Fuente J., Fernández de Mera I.G. (2022). Fatal cases of bovine anaplasmosis in a herd infected with different *Anaplasma marginale* genotypes in southern Spain. Ticks Tick Borne Dis..

[70] Medlock J., Balenghien T., Alten B., Versteirt V., Schaffner F. (2018). Field sampling methods for mosquitoes, sandflies, biting midges and ticks. EFSA Supporting Publicat..

[71] European Centre for Disease Prevention and Control, ECDC Surveillance Systems Overview for 2019. https://www.ecdc.europa.eu/en/publications-data/surveillance-systems-overview-2019.

[72] De Mik E.L., van Pelt W., Docters-van Leeuwen B.D., van der Veen A., Schellekens J.F., Borgdorff M.W. (1997). The geographical distribution of tick bites and *erythema migrans* in general practice in The Netherlands. Int. J. Epidemiol..

[73] Amores Alguacil M., Estévez Reboredo R.M., Martìnez de Aragòn M.V., Carmona R., Cano Portero R. (2022). Carga hospitalaria de enfermedad de Lyme en España (2005–2019). Boletín Epidemiológico Sem..

[74] Ostfeld R.S., Price A., Hornbostel V.L., Benjamin M.A., Keesing F. (2006). Controlling ticks and tick-borne zoonoses with biological and chemical agents. BioScience.

[75] Sánchez-Bayo F. (2021). Indirect effect of pesticides on insects and other arthropods. Toxics.

[76] Marques A.R. (2015). Laboratory diagnosis of Lyme disease: Advances and challenges. Infect. Dis. Clin. North Am..

[77] van der Giessen J., Vlaanderen F., Kortbeek T., Swaan C., van den Kerkhof H., Broens E., Rijks J., Koene M., De Rosa M., Uiterwijk M. (2022). Signalling and responding to zoonotic threats using a One Health approach: A decade of the zoonoses structure in the Netherlands, 2011 to 2021. Euro Surveill..

[78] Garcia-Marti I., Zurita-Milla R., Harms M.G., Swart A. (2018). Using volunteered observations to map human exposure to ticks. Sci. Rep..

[79] Beaujean D.J., Crutzen R., Gassner F., Ameling C., Wong A., van Steenbergen J.E., Ruwaard D. (2016). Comparing the effect of a leaflet and a movie in preventing tick bites and Lyme disease in The Netherlands. BMC Public Health.

[80] Antonise-Kamp L., Beaujean D.J.M.A., Crutzen R., van Steenbergen J.E., Ruwaard D. (2017). Prevention of tick bites: An evaluation of a smartphone app. BMC Infect. Dis..

[81] den Oudendammer W.M., Broerse J.E.W. (2017). Lyme disease in the Dutch policy context: Patient consultation in government research agenda setting. Sci. Public Policy.

[82] Sandberg M., Hesp A., Aenishaenslin C., Bordier M., Bennani H., Bergwerff U., Chantziaras I., De Meneghi D., Ellis-Iversen J., Filippizi M.E. (2021). Assessment of evaluation tools for integrated surveillance of antimicrobial use and resistance based on selected case studies. Front. Vet. Sci.

